# Diagnosis and Treatment of an Ununited Anconeal Process in a California Sea Lion (*Zalophus californianus*)

**DOI:** 10.3390/ani15131865

**Published:** 2025-06-24

**Authors:** Alexander Schlake, Laurens Van Mulders, Moniek Dekkers, Anastasia Selini, Jamie A. MacLaren, Griet Vercauteren, Koen Chiers, Francis Vercammen, Jonas Spruyt

**Affiliations:** 1 Department of Surgery, Clinic for Small Animals, University of Veterinary Medicine Hannover, Foundation, Bünteweg 9, 30459 Hannover, Germany; 2 Small Animal Department, Faculty of Veterinary Medicine, Ghent University, 9820 Merelbeke, Belgium; 3 Centre for Research and Conservation, Royal Zoological Society of Antwerp, 2018 Antwerp, Belgium; 4 Department of Morphology, Imaging, Orthopedics, Rehabilitation and Nutrition, Faculty of Veterinary Medicine, Ghent University, 9820 Merelbeke, Belgium; 5 Functional Morphology Laboratory, Department of Biology, University of Antwerp, 2000 Antwerp, Belgium; 6 Evolution & Diversity Dynamics Laboratory, UR Geology, Université de Liège, 14 Allée du 6 Août, 4000 Liège, Belgium; 7 Palaeobiosphere Evolution Laboratory, Institute of Natural Sciences, Rue Vautier 29, 1000 Brussels, Belgium; 8 Zoolyx NV, Veterinary Laboratory Services, Groeneweg 17, 9320 Erembodegem, Belgium; 9 Pathobiology, Pharmacology and Zoological Medicine, Faculty of Veterinary Medicine, Ghent University, 9820 Merelbeke, Belgium

**Keywords:** California sea lion, *Zalophus californianus*, ununited anconeal process, osteopathology, diagnosis, treatment

## Abstract

This case report describes the first documented occurrence of an ununited anconeal process (UAP) in a California sea lion (*Zalophus californianus*), expanding the species range of this orthopedic pathology beyond domestic canids. A 13-year-old female presented with chronic progressive right forelimb lameness. Diagnostic imaging revealed a large mineralized intra-articular bone fragment at the level of the elbow joint, accompanied by secondary osteoarthritic changes. The fragment was surgically excised via a lateral humero-ulnar arthrotomy. Histologic evaluation of the excised tissue confirmed an ununited ossification center with preserved trabecular architecture and no evidence of inflammation or neoplasia, supporting the diagnosis of UAP. Three-dimensional morphometric analysis demonstrated substantial remodeling of the ulna, humerus, and radius, with morphological deviation in pathological elements exceeding inter-individual variation observed in healthy conspecifics. Despite initial postoperative improvement, the animal succumbed to complications from an unrelated chronic diaphragmatic hernia, four weeks post-surgery. This report introduces UAP as a novel differential diagnosis for forelimb lameness in pinnipeds, and underscores the feasibility of surgical management. The findings provide crucial insights into the comparative pathology related to UAP and orthopedic surgical approaches in marine mammals.

## Simple Summary

Lameness in marine mammals is not well understood, making diagnosis and treatment challenging. This study reports the case of a California sea lion with an ununited anconeal process, a developmental bone condition affecting the elbow joint, similar to a disorder found in domestic dogs. The sea lion had increasing difficulty using its front flipper, and medical treatment did not improve its condition. Surgery was performed to remove the abnormal bone fragment, leading to short-term improvement. Unfortunately, the animal later passed away due to an unrelated medical condition. By studying the removed bone and using advanced imaging techniques, scientists confirmed that the sea lion’s condition was a failure of normal bone development rather than an injury. This finding suggests that similar conditions may exist in other marine mammals but could go unrecognized.

## 1. Introduction

Orthopedic conditions, congenital or acquired, are frequent reasons for presentation of companion animals to veterinarians. Due to their common presentation, a standard protocol for diagnosis and treatment is often in place in many veterinary clinics [1,2]. Extrapolation from companion animal care, especially canines (dogs), is often undertaken by veterinarians when treating exotic animal species [3]; however, this warrants specific understanding of the different physiology and anatomy of each species [4]. Limitations, such as potentially risky anesthesia, limited access to the patient for aftercare, and limited access to specialized equipment in zoological facilities [5], can deter veterinarians from pursuing surgical treatment in those cases, thus presenting a welfare issue for the animals under their care.

The scientific literature on zoo animals with orthopedic conditions, especially marine mammals, mainly focuses on traumatic or acquired conditions rather than congenital diseases [5,6,7,8,9]. Presenting detailed descriptions of successful procedures can enable a more widespread implementation of (orthopedic) surgery for exotic animal species under human care. This is the purpose of the current case report, which provides (to the best of our knowledge) the first description of surgical treatment for a congenital case of an ununited anconeal process in a captive marine mammal: a California sea lion, *Zalophus californianus* (Carnivora: Pinnipedia).

## 2. Materials and Methods

### 2.1. Case Description

A 13-year-old female California sea lion (*Zalophus californianus*), weighing 83 kg and housed at the Royal Zoological Society of Antwerp (RZSA, Antwerp, Belgium), started showing signs of right front limb lameness. Lameness presented as phases of non-weight bearing while standing on land, limping during ambulation, and reduced use of the limb for propulsion while swimming. No trauma was observed, and there was no previous history of lameness. No further medical issues were known at this point. Medical treatments were initiated: 0.25 mg/kg omeprazole (Omeprazole Sandoz^®^ 20 mg/gel, Sandoz, Kopenhagen, Denmark), 0.1 mg/kg meloxicam (Meloxidolor^®^ 5 mg/mL, Le vet Beheer, Oudewater, The Netherlands), 1.2 mg/kg grapiprant (Galliprant^®^ 100 mg/tablet, Elanco, Cuxhaven, Germany), and a dietary supplement containing glucosamine and chondroitin sulphate at 6.0 and 2.3 mg/kg (Seraquin Omega Dog^®^, Boehringer Ingelheim Animal Health, Ingelheim, Germany). The lameness initially improved slightly, but continued to progress over the following three months, necessitating further clinical investigation.

### 2.2. Anesthesia and Diagnostics

#### 2.2.1. First Anesthesia for Radiology

The sea lion was sedated with medetomidine, butorphanol, and midazolam intramuscularly, administered by dart in the left shoulder musculature. Adequate anesthetic depth was attained after 28 min to perform the clinical examination, and the following medical imaging procedures(Table S1).

**Figure 1:**
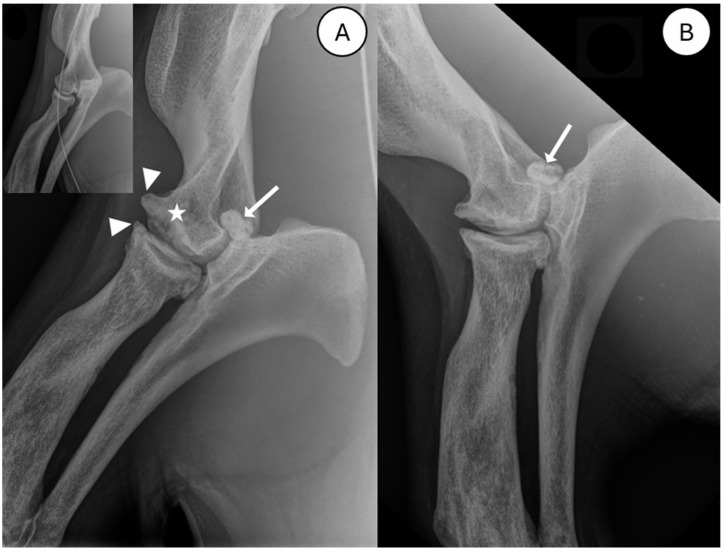
Mediolateral radiographs of the right elbow joint (elbow extended, (**A**); elbow flexed, (**B**)). A large, ovoid, mineralized fragment (indicated by white arrow) is present at the proximal aspect of the anconeal process. An image of the non-pathological left elbow joint, without evidence of an ununited anconeal process, in included for comparison (top left insert); an ECG cable is inadvertently superimposed on this insert image (curved line). White star: concave defect of distal humerus. White triangles: multiple osteophytes.

Digital radiography was performed of the elbow and shoulder joints (left and right). Radiographs were taken with a portable X-ray generator (meX AirRay) with 78 V and 2.5 mAs, captured by a direct detector (Agfa XD 14C) and digitally processed by imaging software (Clearcanvas 13.2). On inspection of the radiographs, a bone fragment was found in the right elbow joint at the location of the anconeal process of the ulna. The anconeal process itself appeared irregularly outlined (Figure 1B), and slightly smaller compared to the contralateral side. The outline of the distal humeral condyle (likely medial) was irregular and flattened, with a concave defect at its distal aspect (Figure 1A, white star). Multiple osteophytes were notably pronounced on the cranial aspect of the distal humeral condyle (Figure 1A, arrows); however, only mild osteophyte formation was present at the cranioproximal aspect of the radius. Additionally, the distal aspect of the radius appeared heterogeneously sclerotic.

Due to progressive lameness and unresponsiveness to medical treatment, a second anesthesia was initiated and surgical removal of the fragment was chosen as the preferred therapy.

#### 2.2.2. Second Anesthesia for Surgery

Forty-six days post-diagnosis, surgery was scheduled. A second anesthesia was initiated with intramuscular sedation via remote dart injection into the left shoulder musculature using medetomidine, butorphanol, and midazolam (Table S2).

After 20 min, a sufficient plane of sedation was reached. A 14 mm endotracheal tube was inserted, and positive pressure ventilation was applied at eight breaths per minute with a tidal volume of 15 mL/kg. Anesthesia was maintained with isoflurane in 100% medical oxygen delivered via a Penlon Prima 440^®^ anesthetic machine. Comprehensive multi-parameter anesthetic monitoring was performed using a uMEC 12 MindRay system, which provided continuous measurements of end-tidal CO_2_ (ETCO_2_), respiratory rate, electrocardiography (ECG), heart rate, pulse oximetry, and esophageal body temperature.

Inhalation anesthesia was discontinued, and the injectable anesthetics, medetomidine and butorphanol, were reversed using atipamezole and naltrexone, respectively. Positive pressure ventilation was maintained until the return of the swallowing reflex, after which the sea lion was extubated once regular spontaneous breathing resumed. The total duration of anesthesia was 110 min from remote dart injection.

### 2.3. Elbow Surgery

#### Surgical Procedure

The sea lion was positioned in sternal recumbency with a slight 10° rotation to the left side, and a slightly caudally extended right front limb for easier access. The surgical site was clipped and shaved, then scrubbed with 40 mg/mL chlorhexidine gluconate mixed 1:1 with tap water, then disinfected with povidone-iodine. Locoregional anesthesia was performed via a brachial plexus block with 4 mg/kg lidocaine diluted 1:1 with sterile saline. The surgical field was sterile draped, and identification of the joint space was undertaken. The very distal part of the tuber olecrani was identified via palpation as the most caudodistal palpable bony prominence at the elbow joint. From there, in a proximal direction, the cranioproximal end of the tuber olecrani was palpated, and ca. 3 cm distal to the former, the extended joint space was palpated.

A skin incision of ca. 7 cm in length with a 10° scalpel blade was made and deepened with monopolar cautery. The subcutis and the prominent subcutaneous fat layer were dissected with Metzenbaum scissors, and the joint space was again palpated. Arthrocentesis was performed, and 1 mL of clear, viscous fluid was aspired and sent for cytology and bacteriology. The humero-ulnar joint space was identified via palpation and slight flexion and extension of the elbow, and the anconeal muscle was bluntly dissected from its humeral insertion. Gelpi retractors were inserted to aid visualization, and arthrotomy with an 11° scalpel blade was performed. The fragment of 0.96 × 1.72 cm diameter with a smooth bony surface was identified further medially in the joint compartment and was still attached distomedially. The fragment was grasped with Mosquito forceps and removed in a twisting motion while cutting with Metzenbaum scissors. The excised fragment was subsequently sent for histology. No further fragments were identified, and the joint capsule was closed utilizing the surrounding muscle fascia with 1 polydioxanone (PDS^®^, Ethicon, NJ, USA) interrupted sutures. The muscle fascia was apposed with 1 polydioxanone in a simple continuous pattern. The deep subcutaneous fat and superficial subcutaneous layers were closed with 2.0 poliglecaprone 25 (Monocryl^®^, Ethicon, NJ, USA) in a simple continuous pattern. The skin was closed with 3.0 poliglecaprone 25 in an intradermal pattern, and ample cyanoacrylate skin glue was applied to the wound. Surgery time was 30 min in total. Postoperative radiographs showed complete removal of the fragment. Cefovecin at 8 mg/kg and 0.5 mg/kg metoclopramide were administered intramuscularly. Postoperative analgesia was continued with 0.1 mg/kg meloxicam in combination with 0.25 mg/kg omeprazole given orally.

After 120 min recovery, access to the enclosure, including the swimming pool, was permitted. Due to the wild character of this animal, it was chosen to not impose postoperative restrictions, i.e., access to the pool.

### 2.4. Necropsy

#### 2.4.1. Post-Surgery Anesthesia for Critical Care

Following observations of anorexia, lethargy, and vomiting, the patient was investigated to assess the condition. An anesthetic procedure was carried out using medetomidine, butorphanol, and midazolam, administered via dart into the shoulder musculature. An adequate plane of anesthesia was achieved after eight minutes, allowing for intubation and the initiation of inhalation anesthesia and perianesthetic monitoring, as previously outlined (Table S3).

Given the compromised condition of the patient, medetomidine was immediately reversed with atipamezole when positive pressure ventilation commenced. Attempts at intravenous cannulation in the interdigital and brachial veins were unsuccessful. The patient experienced cardiac arrest 43 min into the diagnostic procedures (i.e., clinical examination, ultrasonography, and radiography). Emergency interventions comprised the complete reversal of anesthesia using naltrexone and flumazenil administered intramuscularly, along with the administration of atropine and epinephrine via lingual injection. Manual chest compression and positive pressure ventilation were initiated immediately. Despite these resuscitation efforts, which were sustained for 14 min, no cardiac activity was detected, leading to the cessation of resuscitative measures, and the patient ultimately succumbed.

#### 2.4.2. Standard Necropsy

A standard full necropsy was performed in-house at the RZSA for the macroscopic pathologic examination. Representative sections of abnormal bone structures excised from the elbow joint were fixed in a neutral-buffered 10% formalin solution for histological investigation.

### 2.5. Histology

**Figure 2:**
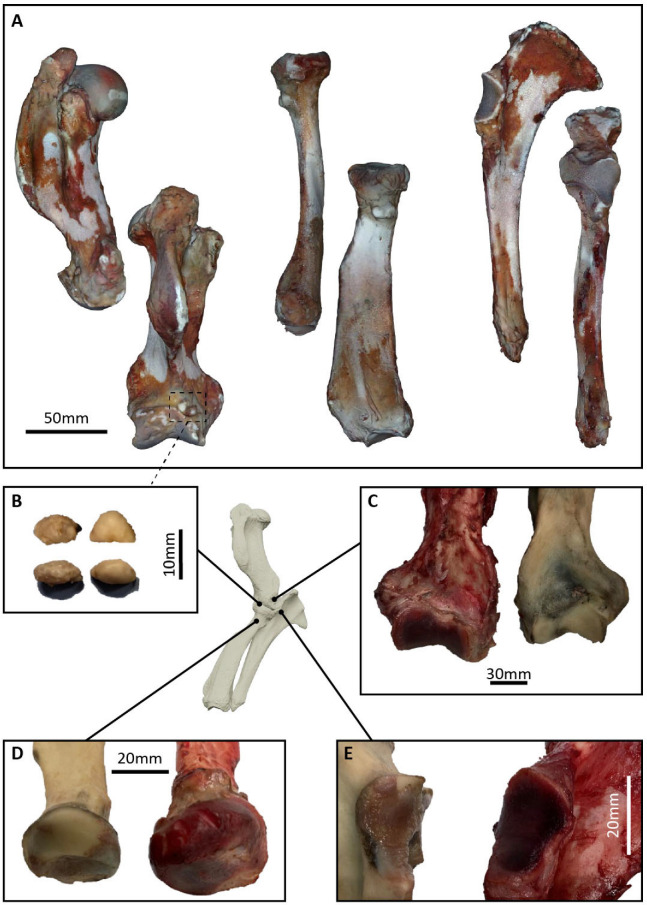
Pathological thoracic limb bones (humerus, radius, ulna) of *Zalophus californianus* in this study. (**A**) Two-dimensional renders of pathological bones (from left): humerus (medial, cranial aspects), radius (lateral, caudal aspects), ulna (medial, cranial aspects). Cranial and caudal aspects show clearly the joint surfaces (humerus, ulna) and bony outgrowth (radius); distal humerus also shows two small bony fragments. (**B**) A 2D image of bony fragments from lateral (top) and dorsal view. (**C**) A 2D image of distal humerus comparison between pathological (left) and healthy bone from the same individual. (**D**) A 2D image of proximal radius comparison between pathological (right) and healthy bone from the same individual, showing clear size discrepancy. (**E**) A 2D image of proximal ulna comparison between pathological (right) and healthy bone from the same individual, showing a clear difference in anconeal and joint surface morphology.

The intra-articular elbow fragment was surgically removed ante-mortem. During the post-mortem, several bone sections were also removed: the proximolateral right radius, the margin of the right olecranon, and the distomedial right humerus. The latter presented with two small, rounded, intra-articular fragments nestled in the coronoid fossa (Figure 2A,B). These bone sections were fixed in formalin, decalcified with formic acid, routinely processed, sectioned, and stained using hematoxylin-eosin. The histological evaluation of the ante- and post-mortem samples were performed by two different board-certified pathologists (Dipl. European College of Veterinary Pathologists [ECVP]).

### 2.6. Three-Dimensional Visualization

Following the sea lion’s demise, her cadaver was inspected via manual dissection at the Veterinary Department of the University of Antwerp. Three forelimb bones constituting the elbow (humerus, radius, ulna) were dissected out from the cadaver and soft tissue material was cleaned off the bone (Figure 2). The humerus, radius, and ulna of both forelimbs (pathological right and non-pathological left) were then scanned using a near-infra-red 3D surface scanner (Moose 3D scanner, 3D MakerPro). Bones were placed on a rotating platform and scanned from multiple sides, producing overlapping point clouds. The point clouds were visualized in JMStudio (JMStudio v.2.6.0, 3D MakerPro); background information was removed manually, and scan alignment was performed. The aligned models were processed and fused to create a closed 3D polygon mesh. The models were imported in Geomagic Wrap (Geomagic Wrap 2017.0.1, 3D Systems Inc., Rock Hill, SC, USA) for a final cleaning step, and filling of holes where necessary (Figure 2). Healthy bones from two other individuals of *Zalophus californianus* previously scanned at the United States National Museum (USNM) using an Artec EVA Lite structured-light scanner (licensed to George Mason University) were used for an inter-individual comparison. To assess the deviation between pathological and non-pathological limb bones, a manual alignment of the 3D scans of pathological and non-pathological bones (inverted to the corresponding orientation) was performed in Geomagic Wrap, allowing inspection of the changes undergone in the pathological elbow bones. Deviations recorded indicated the positional changes between two aligned models, with the cumulative total of positive and negative values equaling the total deviation (difference) between models.

## 3. Results

### 3.1. Radiography

Evaluation of the radiographs of the right elbow revealed a large, ovoid, mineralized intra-articular fragment at the proximal aspect of the right anconeal process. The fragment measured approximately 1.72 cm × 0.96 cm × 1.00 cm. The anconeal process was irregularly marginated at its cranioproximal aspect, and reduced in height compared to the contralateral side (visible in Figure 1). An irregular, convex-shaped distal margin of one of the humeral condyles, most likely the medial condyle, caused malalignment of the humeroradial joint space (Figure 1A). Additionally, there was significant heterogeneous subchondral osteolysis of the distal humerus with adjacent osteosclerosis and marked osteophyte formation at the distal humerus and cranioproximal radius. There was also a heterogeneous osteosclerotic aspect of the radial diaphysis by comparison to the contralateral side. The contralateral limb showed a small osteophyte at the radial head (Figure 1, insert).

### 3.2. Surgical and Post-Surgical Data

#### 3.2.1. Arthrocentesis

The synovial fluid showed slightly increased cellularity with rare erythrocytes on a stippled eosinophilic background. Windrowing was absent, which is indicative of decreased viscosity. The nucleated cell population consisted mainly of large mononuclear cells (72%, macrophages/synoviocytes) and lymphocytes (26%). There were few neutrophils (2%). No microorganisms were found, and aerobic and anaerobic cultures were also negative.

#### 3.2.2. Post-Surgical Evolution

On the first day postoperatively, the animal showed significant lameness on the right front limb in combination with anorexia; the animal was, thus, not compliant with taking any oral medication. However, during the following week, there was gradual improvement in mobility, appetite returned to normal levels, and activity also increased. Two weeks after surgery, lameness was greatly reduced, and mild-to-no lameness was observed in the third and fourth week after surgery; full mobility was, therefore, considered present four weeks post-surgery. These changes were observed without meloxicam. Two weeks post-surgery, signs of wound dehiscence without infection appeared and progressed over several days. By four weeks postoperatively, the wound began to show notable granulation tissue and early epithelialization.

#### 3.2.3. Histology of Intra-Articular Fragment

**Figure 3:**
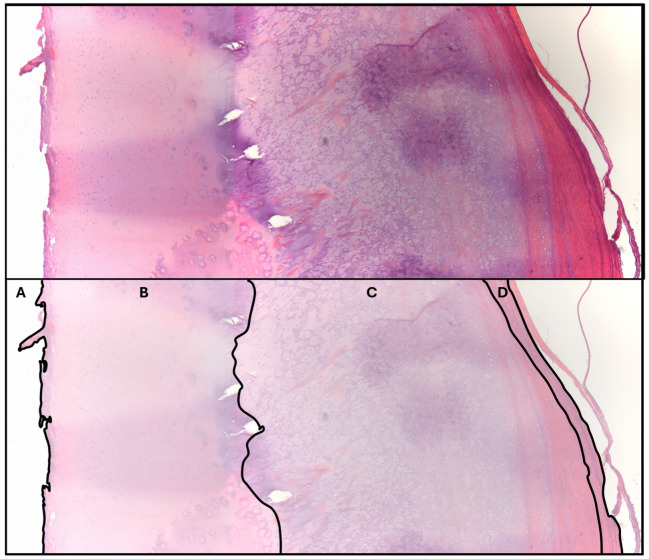
Histological cross-sectional image of the intra-articular fragment. From left to right: (**A**), central empty lumen; (**B**), zone of hyaline cartilage; (**C**), zone of endochondral ossification; and (**D**), small rim of lamellar bone.

At cross section, the fragment was organized in different layers (Figure 3). The center consisted of an irregular empty cavity (Figure 3A), lined by a thick layer of hyaline cartilage (Figure 3B). The cartilage layer included several irregular cavities. The second layer contained an irregular zone of endochondral ossification (Figure 3C); this was finally lined by a thin outer rim of lamellar bone (Figure 3D), demonstrating several clearly denotable cement lines.

### 3.3. Necropsy, Histology, and 3D Visualization

#### 3.3.1. Necropsy

The necropsy revealed a chronic diaphragmatic hernia, approximately 4 cm in diameter, located at the right ventral aspect of the diaphragm. The hernia was characterized by a fibrotic, off-white margin suggestive of longstanding tissue remodeling. The hernia permitted translocation of the entire small intestinal tract into the right hemithorax, resulting in marked thoracoabdominal organ displacement. The stomach exhibited multifocal mucosal ulcerations (ranging from 0.5 cm in diameter to 1 × 4 cm longitudinally) with associated congestion; the cranial duodenum presented with smaller, multifocal ulcerative lesions. The small intestines displayed generalized vascular congestion, and the mesenteric lymph nodes were diffusely enlarged, edematous, and pale. Approximately 50 mL of serohemorrhagic effusion, containing fibrinous material, was present in the peritoneal cavity. The nutritional status of the animal was classified as emaciated; however, inspection of the surgical site on the right flipper revealed a healing superficial wound consistent with the previous orthopedic intervention. As such, no link could be drawn between the forelimb surgical site and diaphragmatic hernia. Gross examination of the right elbow joint revealed marked osteoarthritic changes, including irregular joint surface contours of the distal humerus, proximal radius, and ulna, with visible osteophyte formation and cartilage erosion.

#### 3.3.2. Histopathologic Data

A fragment (1.1 × 1.3 × 0.6 cm) of the pathological proximolateral right radius (Figure 2D) showed almost complete loss of articular cartilage, and replacement with a poorly cellular fibrous tissue. At the margin of the fragment, bordering some strands of dense fibrous tissue (consistent with remnants of capsular or ligamentous insertions), there was osteophyte formation composed of mature bone, and a focus of fibrocartilaginous metaplasia. Subchondral bone was histologically normal. A fragment (1.7 × 1.3 × 0.7 cm) of the margin of the right olecranon (where the anconeal process is normally located) (Figure 2E) was composed of thin, otherwise normal, trabeculae, covered with a cartilage cap of fairly even thickness. The cartilage showed increased matrix eosinophilia, chondrocyte degeneration, and clustering of remaining chondrocytes, most prominently at the base near the subchondral bone. The surface was irregular and focally fissured. A fragment (3.0 × 2.0 × 1.0 cm) of the right distomedial humerus (Figure 2C) was also composed of thin, otherwise normal, trabeculae covered with a cartilage cap. The articular cartilage showed an irregular surface with fissuring. In the more superficial areas, lacunae were often devoid of chondrocytes. There was frequent clustering of remaining chondrocytes. On the right distomedial humerus (coronoid fossa) there were also two, smaller, rounded, intra-articular fragments (0.8 × 0.6 × 0.4 cm) (Figure 2A,B), attached with fibrous tissue to the bone. These fragments were composed of normal trabeculae and bordered by fibrous tissue. In one fragment, the bone was capped by remnants of cartilage and multifocally within the central trabeculae, there was cartilaginous metaplasia.

#### 3.3.3. Three-Dimensional Visualization

**Figure 4:**
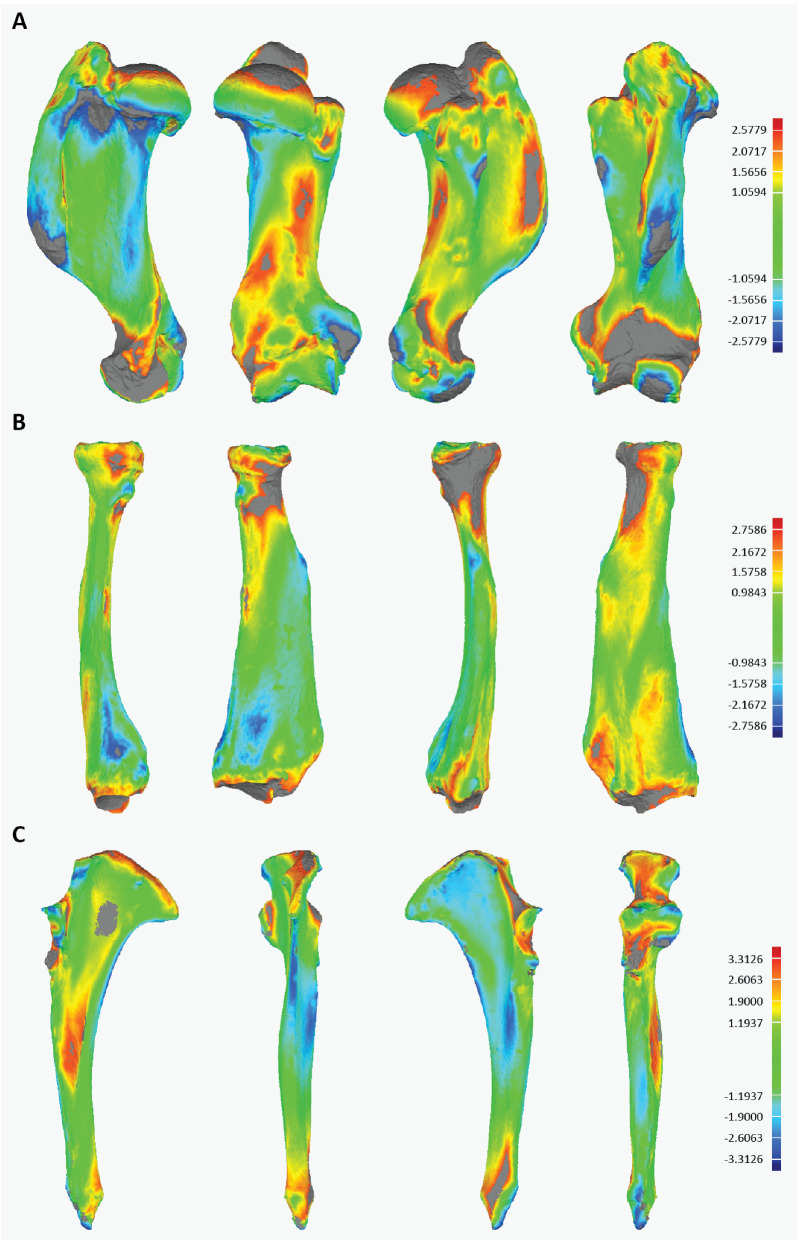
Heatmap visualization of 3D model differences between scanned pathological and non-pathological *Zalophus californianus* limb elements. Limb elements are shown alongside scale of deviation between models (in mm): (**A**) humerus, (**B**) radius, (**C**) ulna. Warm and cool colors highlight regions of the bone that deviate most greatly between healthy and pathological specimens; areas in grey denote regions so far deviated that they are outside the range of detection. From left: lateral view, caudal view, medial view, cranial/dorsal view.

The 3D visualization of the bones of the front limbs of the sea lion demonstrated clearly the differences between healthy and pathological bones, particularly the joint surfaces around the distal humerus (Figure 4A) and proximal radius and ulna (Figure 4B,C). The 3D model surfaces highlighted in grey marked morphologies beyond the range of detection by the alignment algorithm; these regions were the most heavily divergent between the models. Areas of highest divergence, therefore, included the medial region of the head of the radius and the trochlear fossa of the humerus (Figure 4A,B). Highest maximal deviations detectable (6.624 mm) and average deviations (1.19 mm) were recorded in the ulna (Figure 4C; Table 1). The humerus and radius maximal deviations both exceeded 4.0mm, with average deviations around 1.0 mm (Figure 4A,B; Table 1). Table 1 also shows the dimensions of healthy bones from the subject animal and two other individuals of *Zalophus californianus* from the USNM.

All 3D scans associated with this study are available on morphosource.org, accessed 3 January 2025, project ID: 000697545.

## 4. Discussion

The current report on a novel surgical removal of an ununited anconeal process (UAP) in a California sea lion details a successful approach to treating this condition in a non-companion mammal species. Despite the subsequent death of the individual from an unrelated diaphragmatic hernia, the surgical treatment of the UAP in this individual (and three-to-four-week postoperative period) lead to a full recovery of forelimb mobility. A bacterial culture of the elbow synovial fluid and the cytological findings suggest a non-inflammatory and non-infectious arthropathy. These conditions are often degenerative in origin. Furthermore, the bone fragments examined from the right elbow showed gross and histologic lesions consistent with degenerative joint disease. Cartilage degeneration and osteophyte formation were the most prominent changes. The right limb processus anconeus of the ulna showed similar cartilage abnormalities as those found on the distal humerus and right proximal radius of the same limb. Based on their histological characteristics, we hypothesize that the two smaller fragments found within the joint capsule (Figure 2B) were most likely released osteophytes.

All findings indicate the presence of a large, loose, mineralized osteophyte and marked secondary osteoarthritis of the elbow joint. Histological examination of this fragment showed normal ossification, without signs of inflammation or neoplasia. The anconeal process (or possibly the distal humeral condyle) was considered as a possible location of origin of the mineralized fragment; this could have been due to elbow dysplasia with either a UAP or osteochondrosis dissecans (OCD), or due to trauma. The medial attachment of the excised fragment to the anconeal process, along with the adjacent abnormal morphology of the anconeal process itself, suggests that the origin of the fragment is most likely a UAP. Histological analysis further supports this diagnosis, since normal ossification was identified with normal trabeculae, indicating an ununited ossification center as the most probable cause. In the case of OCD, the expected histological findings would include disruption of endochondral ossification, alterations in subchondral bone, and abnormal cartilage, characterized by areas of necrosis and fibrillation [10]. A traumatic origin is also considered less likely, as it would typically present with histological evidence of callus formation or other signs of bone healing. Radial diaphyseal osteosclerosis was considered due to abnormal usage of the limb.

When healthy bones from the sea lion of the Royal Zoological Society of Antwerp (RZSA) were compared in the same way to healthy bones from other individuals of *Zalophus californianus* from the United States National Museum (USNM, Washington, DC, USA), deviations between bones of two different individuals were higher than those between pathological and healthy bones for the humerus. By contrast, deviations were similar in magnitude between the two comparative samples for the radius, with inter-individual differences slightly lower than healthy-vs.-pathological differences. The ulna was notably more divergent in shape in the healthy-vs.-pathological comparison than in the inter-individual comparison. Deviations between the joint surface and epicondylar morphology highlight the functional impact that this pathology had on the elbow joint. Overall, the ulna was remodeled most extensively of the stylopodial and zeugopodial limb elements, despite extreme localized remodeling in the distal humerus and proximal radius.

UAP is a well-described condition affecting dogs around five months of age, especially in shepherd breeds and other large-breed dogs [11,12]. Traditionally, UAP has been described as a failure of the fourth ossification center of the ulna to unite with the ulnar metaphysis [13]. Several different hypotheses regarding the cause of UAP have been reported, including asynchronous growth of radius and ulna causing pressure on the anconeal ossification center and delayed or no fusion, underdevelopment of trochlear notch, metabolic disturbances, repeated growth plate trauma, delayed endochondral ossification, and genetic components [11,12,13,14,15]. Since the origin of pinnipeds from arctoid carnivorans (within the Caniformia) is well supported [16], a shared predisposition for certain genetically influenced congenital diseases between canids (e.g., dogs) and pinnipeds (e.g., seals and sea lions) may be present. However, despite a relatively regular occurrence (0.6%) in domestic dogs [17,18], reports of ununited anconeal processes in other species of caniforms are not present to date, and a literature search on PubMed before the surgery and during the writing of this report revealed no publications on ununited anconeal process pathology other than in dogs. This is not to say that the condition is not prevalent in other caniform taxa (wild or in captivity), but simply that it has not been reported. Future observations of forelimb immobility or lameness in captive mammals (particularly caniforms) may build upon this occurrence information, which at present remains restricted to only *Canis* and *Zalophus* genera.

Access to the elbow joint in the sea lion was different compared to the dog: the lateral epicondyle serves as an anatomical landmark for orientation in the dog, but was not palpable in the sea lion [13]. Instead, the distal part of the tuber olecrani was easy to palpate and was used as the first landmark. The more proximal “hook” on the proximal part of the tuber olecrani served as a second landmark, from which a perpendicular line to the cranial outline of the flipper was chosen based on the radiograph. The location of the humero-ulnar joint was located via arthrocentesis, or by the visible elevation caused by accumulation of joint fluid in the patient. Access to the intra-articular fragment was quick; however, the joint space was much deeper in a mediolateral direction compared to the dog. The fragment, compared to that than usually seen in dogs, was not heavily attached to surrounding tissue and, due to the space available, was much easier to remove. In dogs, arthrotomy for UAP removal is often undertaken after arthroscopy to confirm diagnosis and to check for other features of elbow dysplasia [19]. Arthroscopy was not undertaken in this case due to the unavailability of equipment and potentially prolonged anesthesia time.

The closure of the wound was performed in a similar manner to the dog, except for an additional subcutaneous fat layer closure, which is much more prominent in pinnipeds. Meticulous closure was attempted, as no exercise restriction was possible and immediate release back into the pool potentially increased the risk of dehiscence and infection. However, there was limited concern for the healing of a small surgical wound, given the remarkable wound healing capacities in pinnipeds [8,20], e.g., as shown after shark bites [21]. As an additional possible countermeasure for postoperative infection, the salinity in the pool was increased immediately postoperatively to 35 ppt.

In dogs, there is often a long recovery period of multiple months after UAP surgery, both due to the invasive nature of the surgery and the underlying condition causing the UAP generation [13]. The sea lion showed some marked improvement within 4 weeks, walking and swimming nearly free of visible lameness at the end. This might be due to the anatomical difference to dogs, because the ulna does not appear to be a weight-bearing surface during standing and movement, but rather stabilizes the humero-radial joint against lateral movement translation [22]. This might also explain the late onset of clinical symptoms compared to dogs, assuming this was an example of a congenital or early-onset condition, rather than an acquired condition.

While the short-term outcome regarding usage of the limb was very positive, we were unfortunately not able to draw long-term conclusions about the success of the treatment. The osteoarthritis of the radius and humerus visible on the preoperative radiographs, in this case, is likely secondary to the UAP fragment, and may have progressed in the future even without the inciting cause being present anymore. However, radiological findings of osteoarthritis do not necessarily correlate with clinical lameness and pain in other species; therefore, long-term freedom of clinical symptoms appears possible [23,24]. The remodeling of the bone around the elbow joint in the pathological bones represented morphological modification which exceeded the differences measured between healthy bones of two distinct individuals of *Z. californianus*. This demonstrates a substantial degree of bone remodeling in the pathological stylopodial and zeugopodial elements, although this did not manifest in right- (or left) side dominant locomotion post-injury or post-surgery. At this point, no quantitative functional conclusions may be drawn on any restrictions of locomotor efficiency (either in aquatic or terrestrial movements). The collection of 3D scans post-mortem may open up interesting research avenues to pursue in the future; additionally, performing such scans in other individuals suspected of suffering from UAP (domestic or captive wild animals) may provide more information on the types of remodeling which can be associated with this condition.

While the diaphragmatic hernia diagnosed at necropsy does not appear to be caused by surgery or general anesthesia, the question of its origin remains. While acute trauma was not observed, blunt force trauma is one of the known causes and interaction with one of the other sea lions may be one of the possibilities. Congenital diaphragmatic hernia is a known condition in other species, albeit rarely described in pinnipeds [25]. The herniated diaphragm was likely the cause of clinical deterioration and perianesthetic death. It may have worsened due to perianesthetic events, ultimately causing cardiovascular failure during the last procedure. Preoperative radiographs may have been useful in diagnosing this condition; however, no clinical signs were present, and the prevalence in this species has not been reported.

Unfortunately, no good long-term outcome for this patient could be achieved due to unrelated pathology causing the death of the patient. However, after carefully considering their unique anatomy and physiology, we believe the initial response to surgical treatment warrants reporting for future attempts at orthopedic surgery in pinnipeds, investigations into UAP prevalence, and may be added to the best-practice guidelines for treatment of these charismatic marine mammals [26].

## 5. Conclusions

This case represents the first report with conclusive evidence of an ununited anconeal process in a non-canid species, with comprehensive support from radiology, cytology, histopathology, and 3D morphometry. This condition should be considered as a differential diagnosis to OCD or trauma for forelimb lameness in pinnipeds. As otariid pinnipeds (= sea lions and fur seals) are regularly housed as crowd-pleasing additions to zoological parks and aquaria, this finding may have widespread applications in other captive circumstances. Additionally, the surgical treatment for this condition thoroughly described here may serve as a more effective alternative to medical treatment in similar cases in the future.

## Figures and Tables

**Table 1:** Deviation values for humerus, radius, and ulna 3D scans comparing pathological and healthy limb bones, and comparisons between healthy bones from subject animal and other specimen(s) of *Zalophus californianus* from USNM collections. Maximum deviation, average deviation, and standard deviation are presented.

Comparison	Maximum Deviation (mm)	Average Deviation (mm)	Standard Deviation (mm)
Humerus			
RZSA healthy vs. RZSA pathological	2.578	1.059	0.673
RZSA healthy vs. USNM 200847	2.821	1.138	0.772
Radius			
RZSA healthy vs. RZSA pathological	2.758	0.984	0.656
RZSA healthy vs. USNM 21735	2.535	0.902	0.677
Ulna			
RZSA healthy vs. RZSA pathological	3.313	1.194	0.822
RZSA healthy vs. USNM 21735	3.078	0.879	0.737

## Data Availability

The original contributions presented in this study are included in the article/Supplementary Material. Further inquiries can be directed to the corresponding author.

## Supplementary Materials

The following supporting information can be downloaded at: https://www.mdpi.com/article/10.3390/ani15131865/s1, Table S1. First Anesthesia for Radiology. Table S2. Second Anesthesia for Surgery. Table S3. Third Anesthesia for Critical Care.
