# Supplementary material for: Diagnosis and Treatment of an Ununited Anconeal Process in a California Sea Lion (Zalophus californianus)
Source: Animals (Basel). 2025 Jun 24;15(13):1865. doi: 10.3390/ani15131865 (PMC12249024; doi:10.3390/ani15131865)
Supplement: Supplementary file 1 [file animals-15-01865-s001.zip › animals-3613856-supplementary.pdf]

Table S1. First Anesthesia for Radiology

Drug	Commercial name, manufacturer	Dose	Route of administration	Time to onset
Medetomidine	DorbeneVet®, Laboratories Syva, Spain	0.02 mg/kg	Intramuscular	28min
Butorphanol	Butomidor®, Vetviva Richter, Spain	0.2 mg/kg	Intramuscular	28min
Midazolam	Dormazolam®, Le vet Beheer, Netherlands	0.2 mg/kg	Intramuscular	28min

The sea lion was sedated with medetomidine, butorphanol and midazolam intramuscularly, administered by dart in the left shoulder musculature. Adequate anesthetic depth was attained after 28 minutes to perform the clinical examination and the medical imaging procedures.

Table S2. Second Anesthesia for Surgery

Drug	Commercial name, manufacturer	Dose	Route of administration	Time to onset for i.m. sedation
Medetomidine	DorbeneVet®, Laboratories Syva, Spain	0.03 mg/kg	Intramuscular	20min
Butorphanol	Butomidor®, Vetviva Richter, Spain	0.2 mg/kg	Intramuscular	20min
Midazolam	Dormazolam®, Le vet Beheer, Netherlands	0.2 mg/kg	Intramuscular	20min
Isoflurane	Isoflutek®, Laboratorios Karizoo, Spain	0.5-1.5%	Inhalation	
Atipamezole	Alzane®, Labroatorios Syva, Spain	0.15 mg/kg	Intravenous	
Naltrexone	Trexonil®, Wildlife Pharmaceuticals, South Africa	0.2 mg/kg	Intravenous	

A second anesthesia was initiated with intramuscular sedation via remote dart injection into the left shoulder musculature using medetomidine, butorphanol and midazolam. After 20 minutes, a sufficient plane of sedation was reached, a 14 mm endotracheal tube was placed, and positive pressure ventilation was applied at 8 breaths per minute with a tidal volume of 15 ml/kg. Anesthesia was maintained with isoflurane in 100% medical oxygen delivered via a Penlon Prima 440® anesthetic machine. Comprehensive multi-parameter anesthetic monitoring was performed using a uMEC 12 MindRay system, which provided continuous measurements of end-tidal CO_2 ($ETCO_2$), respiratory rate, electrocardiography (ECG), heart rate, pulse oximetry, and esophageal body temperature.
Inhalation anesthesia was discontinued, and the injectable anesthetics, medetomidine and butorphanol, were reversed using atipamezole and naltrexone, respectively. Positive pressure ventilation was maintained until the return of the swallowing reflex, after which the sea lion was extubated once regular spontaneous breathing resumed. The total duration of anesthesia was 110 minutes from remote dart injection.

Table S3. Third Anesthesia for Critical Care

Drug	Commercial name, manufacturer	Dose	Route of administration	Time to onset for i.m. sedation
Medetomidine	DorbeneVet®, Laboratories Syva, Spain	0.02 mg/kg	Intramuscular	8min
Butorphanol	Butomidor®, Vetviva Richter, Spain	0.2 mg/kg	Intramuscular	8min
Midazolam	Dormazolam®, Le vet Beheer, Netherlands	0.2 mg/kg	Intramuscular	8min
Atipamezole	Alzane®, Labroatorios Syva, Spain	0.1 mg/kg	Intravenous	
Naltrexone	Trexonil®, Wildlife Pharmaceuticals, South Africa	0.4 mg/kg	Intramuscular	
Flumazenil	Anexate®, Cheplafarm, Germany	0.025 mg/kg	Intramuscular	
Atropine	Atropine Sulphate Sterop®, Laboratoires Sterop, Belgium	0.05 mg/kg	Lingual injection	
Epinephrine	Adrenaline Sterop®, Laboratoires Sterop, Belgium	0.1 mg/kg	Lingual injection	

Anesthetic procedure was carried out using medetomidine, butorphanol and midazolam, administered via dart into the shoulder musculature. An adequate plane of anesthesia was achieved after 8 minutes, allowing for intubation and the initiation of inhalation anesthesia and perianesthetic monitoring, as previously outlined. Given the patient's compromised condition, medetomidine was immediately reversed with atipamezole when positive pressure ventilation commenced. Attempts at intravenous cannulation in the interdigital and brachial veins were unsuccessful. The patient experienced cardiac arrest 43 minutes into the diagnostic procedures, i.e., clinical examination, ultrasonography, and radiography. Emergency interventions comprised the complete reversal of anesthesia using naltrexone and flumazenil administered intramuscularly, along with the administration of atropine and epinephrine via lingual injection. Manual chest compression and positive pressure ventilation were initiated immediately. Despite these resuscitation efforts, which were sustained for 14 minutes, no cardiac activity was detected, leading to the cessation of resuscitative measures, and the patient ultimately succumbed.
